# Creating climate-informed physician leaders: The evolution of a physician fellowship in climate and health science policy

**DOI:** 10.3389/fmed.2022.1060145

**Published:** 2022-12-20

**Authors:** Stefan Wheat, Bhargavi Chekuri, Cecilia Sorensen, Rosemary Rochford, Jay Lemery

**Affiliations:** ^1^Department of Emergency Medicine, School of Medicine, University of Washington, Seattle, WA, United States; ^2^Climate and Health Program, University of Colorado School of Medicine, Aurora, CO, United States; ^3^Department of Environmental Health Sciences, Mailman School of Public Health, Columbia University, New York, NY, United States

**Keywords:** climate health, medical education, climate change, health literacy, planetary health, health systems, health policy

## Abstract

Climate change poses numerous near and long-term challenges for our society, and the human health consequences are increasingly recognized as unprecedented. Responding to these health hazards requires a healthcare workforce composed of climate-informed clinicians. As trusted messengers, physicians play a vital role in informing and preparing the public for health impacts of climate change. We describe an evolving graduate medical education fellowship for physicians from all specialties capable of training leaders in this field. Our program pairs fellows with federal and non-governmental partners to provide expertise in climate policy and empower them to be change agents. The accelerating response to climate change from the federal government coupled with an increased recognition of the impacts of climate hazards on health demands a climate-informed clinical workforce. The expansion of this fellowship to accommodate trainees from multiple specialties and its innovative structure leveraging local and national partnerships sets a standard for how similar programs can be developed in addressing the greatest public health threat and opportunity of the century.

## 1 Introduction

The effects of climate change on humans around the world has never been more apparent. Climate related extreme weather events serve as stark reminders that the IPCC’s forecasting of “unprecedented” impacts from climate change is no idle warning (1). An editorial published in 200 international health journals including the Lancet and the New England Journal of Medicine has identified climate change as the “greatest threat to global public health” and serves as a call to arms for health professionals worldwide (2, 3).

Domestically, the National Academy of Medicine have launched a *Grand Challenge of Climate Change and Human Health*, explicitly identifying education about “the climate crisis as a public health and equity crisis” as one of its strategic objectives (4). Further, the Biden administration has placed climate and health at the forefront of its policy agenda, focusing specifically on health equity as it applies to climate change (5).

Direct and indirect health effects expected as a result of climate change include higher rates of respiratory and heat-related illness, increased prevalence and varying distribution of vector-borne diseases, increased food insecurity and malnutrition, and worsening mental health problems (6). Additionally, the increased intensity, severity, and frequency of extreme weather events driven by climate change will compromise access to care for patients and the overall quality of care providers are able to provide (7). Perhaps most importantly, the health effects of climate change will amplify healthcare disparities along racial, ethnic, gender and socioeconomic lines and thereby exacerbate existing healthy and social inequities (8). The process of building climate resilient health systems must be informed by and take into consideration local structures of health-care governance and implementation while also involving stakeholders, including physicians who can guide the process and ensure a safe and patient-oriented transition.

Physicians, by virtue of their training and practice, are particularly well positioned to address issues at the intersection of climate change and health equity. Recognizing the need and opportunity posed by the health impacts of climate change, in 2017 the University of Colorado School of Medicine (CU SOM) developed a novel graduate medical education (GME) fellowship in Climate and Health Science Policy, designed to train a new generation of physician leaders equipped with the scientific background, educational knowhow, leadership, organizational, and advocacy skills to tackle this far-reaching crisis (9). The original cohort of fellows were emergency medicine physicians. Working off the success of the initial cohort, the fellowship was expanded in 2021 to encompass trainees from all medical backgrounds. In this paper, we describe the evolution of this fellowship, expanded to greater scale and with the goal of training across all medical specialties.

### 1.1 Problem identification and needs assessment

Historically, the climate crisis has been framed as an environmental challenge and health impacts have been a footnote. Recent literature highlights the increasingly important role of health as a lens through which climate impacts can be made more salient and tangible for the public and policymakers (10). Health is greatly impacted by factors beyond healthcare and therefore health policy must be addressed through a broad, cross-sectoral approach. This is acknowledged by *Health-in-All* initiatives espoused by leading public health organizations like the Centers for Disease Control and Prevention (CDC) and the World Health Organization (WHO) (11).

Physicians continue to act as trusted public health communicators and drivers of policy (12). However, a workforce of clinicians with the skills to address the climate crisis remains conspicuously absent, despite their potentially critical role at the nexus of climate and health policy and the increasing demand that climate and health education be integrated into health curricula (13, 14). Although some institutions have started integrating climate and health into medical student education, they represent outliers (15). A study in 2020 found that only 15% of 2817 medical schools across 112 countries offer any curricula on climate change and health (16).

The *Lancet* has identified climate change as “the greatest global health threat (and opportunity) of the 21st century (17).” Within this context, there is a vital need for leaders who are equipped with the skills and experience to effectively operate and provide critical analysis in the climate and health space. While the movement to engage physicians on the topic of climate and health remains a nascent one, the opportunity for physician led climate-health policy is great. This is fundamentally an issue of an impending workforce shortage. We discuss a fellowship capable of filling in this gap.

### 1.2 Expansion of prior model

In response to the growing need for healthcare professionals capable of contextualizing climate change with health, faculty in the Department of Emergency Medicine at the University of Colorado established the first non-accredited graduate medical education (GME) fellowship in climate and health science policy in 2017. The pilot model allowed for one board eligible or certified emergency medicine physician to be integrated into the UCHealth System as a GME fellow who could offset his/her fellowship salary through clinical shifts as a practicing attending physician. The fellow’s remaining time was spent completing non-clinical activities at federal agencies and with various non-governmental organizations (NGOs). This was a successful model with prior fellows going on to be selected for national leadership positions in climate and health, presenting at national conferences, and authoring original peer-reviewed publications among other honors.

## 2 Pedagogical framework

### 2.1 Overall structure educational strategies and implementation

The foundation of the Fellowship rests on four pillars, with the goal of training physicians proficient and credible in climate and health science capable of assuming leadership positions, disseminating knowledge and influencing policy:

#### 2.1.1 Advocacy and leadership

Fellows become fluent in effective advocacy and leadership skills while partnering with NGOs including Healthcare Without Harm (HCWH), ecoAmerica, The Nature Conservancy, the National Resources Defense Council (NRDC), the Payne Institute of Public Policy, the Global Consortium of Climate Health Education (GCCHE) and others.

#### 2.1.2 Clinical practice and professional development

Fellows are all practicing clinicians from diverse backgrounds, specialties, and geographic locations. Clinical practice provides fellows unique expertise while working with their federal and NGO partners. In addition, they learn how to integrate climate and health education into their individual specialties and careers and are empowered to influence organized medicine and their respective academic medical departments to engage with this burgeoning field.

#### 2.1.3 Education and academic development

Fellows receive and participate in a variety of activities including weekly didactics from core and guest faculty as well as teaching and research opportunities which foster academic development, innovation, and scholarly productivity in climate and health education.

#### 2.1.4 Policy expertise

Fellows gain policy expertise with placements in major federal agencies including the Environmental Protection Agency (EPA), the U.S. Global Change Research Program (USGCRP), the National Oceanic and Atmospheric Administration (NOAA), the CDC, and OCCHE.

### 2.2 Advocacy and leadership

Fellows engage in a variety of activities to develop leadership and capacity to advocate for health system action and cross-sectoral policy solutions to address local, federal and global health opportunities related to climate change. All fellows participate in advocacy workshops at CU SOM, where they learn and practice how to effectively communicate with elected officials as well as testify at legislative hearings. Additionally, they have longitudinal partnerships with NGOs like GCCHE, HCWH, EDF, the Payne Institute of Public Policy and EcoAmerica. While working with these organizations, fellows become fluent in utilizing multi-modal strategies to engage the public and other physicians and leaders on issues at the intersection of climate and health.

### 2.3 Clinical practice and professional development

Clinical practice differentiates fellows from many other practitioners in the field of climate and health traditionally dominated by public policy and public health experts. Directly engaging with patients impacted by climate change in their day-to-day practice provides a unique perspective that lends credibility to fellows in their advocacy and education efforts.

Having fellows in multiple specialties has meant being thoughtful about mentorship on how fellows can integrate climate and health education, advocacy and leadership into their respective fields and careers. Recognizing that climate-informed healthcare is a novel, but burgeoning field in many specialties, there has been particular focus among fellowship faculty to provide meaningful mentorship on this front. As a fellowship that is not tied to a certificate or degree, fellows must consider factors that will facilitate their marketability beyond the fellowship. Fellows meet with faculty on a monthly basis to discuss professional development, as well as to track progress on deliverables to ensure they are all well positioned to pursue leadership positions post-fellowship.

### 2.4 Education and academic development

Core objectives of the fellowship are to gain understanding of the complex relationships between climate change and health as well as assess population-based climate hazards and analyze public health interventions. Fellows participate in a weekly didactic, administered over a live, virtual platform in which subject-matter experts teach the fundamentals of climate and health science, public health impacts and interventions as well as core concepts in health policy and health science communication. Bolstered by a robust community of practice amongst their co-fellows, fellows learn from one another and benefit from educational opportunities fostered by their peers.

Another core objective of the fellowship is to foster an environment where fellows become effective educators. Development of skills in education and academics is facilitated through many opportunities for teaching at CU SOM and other institutions. All fellows help administer and teach a fourth-year medical school elective course in Climate Medicine at CU SOM. Based on personal interest, they also participate in continuous evaluation of curricular design for this course while simultaneously gaining expertise in academic innovation in climate change and health. Lastly, all fellows help build a forthcoming 300-h professional development course for clinicians titled *Diploma in Climate Medicine* at CU SOM.

Fellows further engage with their NGO partners in educational programs such as collaboration with Project ECHO’s Climate Change and Human Health interactive webinar series, development of curricula with GCCHE and efforts to develop a novel *Climate and Health Responders Course* in collaboration with NOAA and Project ECHO.

Academic development for fellows is further supported with ample opportunities for research and scholarly productivity. Fellows develop a network of mentors with their federal and NGO partners tailored to suit their particular research interests.

### 2.5 Policy expertise

Fellows benefit from preceptorships at one of the five major federal agencies listed above working on climate and health issues. These preceptorships supply critical insight into real-time developments in climate and health research, policy, and advocacy. Further, they allow fellows to gain experience navigating the unique challenges that arise with federal planning and program implementation. Fellows communicate directly with leadership at their federal placements and external stakeholders, help draft internal, interagency, and public communication materials, and contribute to academic papers, federal reports and products such as the National Climate Assessment. They also have opportunities to learn how various federal funding streams are allocated by federal agencies while investing in climate and health research. During all these activities, fellows offer their valuable expertise as practicing clinicians and “end users.”

Fellows also have the opportunity to attend national and international conferences, including the United Nations Framework Convention of Climate Change’s (UNFCCC) Conference of Parties (COP) where they are able to see first-hand how global climate policy is negotiated, as well as collaborate with and engage with leading global climate and health organizations, including the WHO. With the overarching goal of producing climate-informed physician leaders in healthcare, health policy and medical education, we established the competencies detailed in Table 1.

**TABLE 1:** Set of competencies for the climate change and human health physician fellowship.

**● Competency #1**: Fundamentals of climate and health–Fellows will gain an understanding of the complex relationships between climate change and health.
**● Competency #2**: Climate change and public health–Fellows will demonstrate competence in assessing population-based climate hazards and analyzing public health interventions.
**● Competency #3**: Climate change and clinical practice–Fellows will understand the clinical and health care system impacts of climate change and demonstrate competence with the recognition and management of patient and health care system vulnerabilities.
**● Competency #4**: Policy aspects of climate change and health–Fellows will demonstrate familiarity with international and domestic policies and advocacy relevant to climate change and health.
**● Competency #5**: Communication and leadership–Fellows will demonstrate competence in effective climate and health communication and education among different audiences as well as gain insight into how to effectively lead health programmatic development within academic, public and private sectors.

## 3 Learning environment

With the success of the pilot cohorts, the opportunity to build a larger community of climate-informed physicians across all specialties was realized. Moreover, the mainstream adoption of the virtual classroom and workplace during the COVID-19 pandemic facilitated expansion of the prior model to a national fellowship model, hosting five fellows in various medical specialties. The fellowship is hosted at CU SOM where fellows are given an academic home and act as visiting scholars, participating in the core, non-clinical components of the fellowship virtually. In parallel, fellows are given the liberty to work clinically at any location or institution of their choosing in the US. While the original non-accredited GME fellowship position has been retained, with one fellow continuing to practice clinically at UCHealth, the expansion of the fellowship has allowed for significant flexibility in number, type, and location of applicants and fellows.

### 3.1 Duration

The duration of the fellowship is 1 year. The fellow in residence at the University of Colorado Department of Emergency Medicine has the option, in consultation with program leadership, to extend the fellowship to 2 years for completion of the fellowship with concomitant degree-seeking coursework such as a Master of Public Health.

### 3.2 Fellowship administration

The fellowship is administered through the Dean’s Office at CU SOM with administrative support through the Department of Emergency Medicine, Section of Wilderness and Environmental Medicine (WEM). The fellowship director is clinical faculty at CU SOM. Site preceptors at federal and NGO partner institutions work individually with fellows to arrange for training deployments for virtual or in-person collaboration on a case-by-case basis. The Section of WEM provides administrative support for the fellowship.

### 3.3 Faculty and advisory committee

Fellowship faculty assist the fellow through scholarship and research mentorship, through access to meetings and collaborative policy working groups (government, private, and public sector), and through diverse expertise and connectivity. Advisory committee members represent basic sciences and clinical departments within the CU SOM, Colorado School of Public Health, and government entities such as the CDC and HHS.

### 3.4 Evaluation and feedback

The fellow has formative quarterly meetings with program faculty that incorporate feedback from site preceptors and serve as formal assessments on meeting Fellowship goals, competencies and objectives. A summative assessment of both the individual Fellow and the overall Fellowship is submitted to the Dean and the Fellowship advisory committee by the fellowship director annually. The summative assessment incorporates quantitative survey data sent to fellowship principals and site preceptors, as well as qualitative feedback.

The Fellow is likewise mandated to provide formative and summative assessment on congruence with the fellowship experience and the “boots on the ground” reality with stated goals and objectives. Given the fast-paced and dynamic nature of this Fellowship, we have also established a culture of less formal, “just-in-time” feedback from the Fellow to program faculty.

## 4 Assessment

Capacity for formal evaluation of fellowship participants was limited due to the initial skeleton faculty involved in fellowship administration. Previous evaluation of fellow performance and feedback from the fellows on their experience with the fellowship has taken the form of informal conversations with fellowship leadership. There is a plan to transition to more formal evaluation methods with quantitative and qualitative surveys. Future fellowship participants will undergo formal exit interviews to capture a more holistic program evaluation. Evaluation will be three-pronged and include program evaluation tools (e.g., review of fellowship goals and objectives for participant relevance, review of teaching quality), process evaluation tools (e.g., questionnaires to assess participant experience, interviews to assess learning environments), and participant evaluation tools (e.g., formal feedback sessions for students and faculty, peer evaluation, etc.). These steps are meant to improve the fellowship’s capacity for process improvement from year to year. At present, the fellowship has contributed to the training of 13 GME fellows from a range of medical specialties including family medicine, internal medicine, emergency medicine, neurosurgery, and neurology. 10 out of the 13 fellows to date have identified as female while 3 have identified as male. Fellows have varied in career stage from early career professional to mid-career professionals. In addition, all fellows participating in the fellowship thus far have been clinically active in their chosen specialties during the course of their participation with the fellowship.

Previously, fellowship assessment has been defined by deliverables contributed by fellows in their various relationships with federal preceptors, non-profits, and fellowship faculty. Past fellow scholarship has ranged from primary research, policy papers, op-eds, conference proceedings, and systematic review articles. Examples include: Technical contributor to 2019 Lancet Countdown on Health and Climate Change, Technical contributor for 4th National Climate Assessment, Co-editor on a major climate health textbook for health providers and policymakers titled *Global Climate Change and Human Health: From Science to Practice*, Co-author on a paper defining the link between women’s health and climate change, Co-author of landmark public health research study in Puerto Rico: *Mortality in Puerto Rico After Hurricane Maria.*

## 5 Discussion and future directions

This past year, we have seen unprecedented kinetic activity and commitment across many sectors toward climate and health activities: global governance; changes in funding priorities; healthcare systems; medical education and U.S government policy. Our program meets these tremendous opportunities by training credible, knowledgeable, and effective leaders in climate and health. To date, our Fellows hail from the specialties of family medicine, emergency medicine, and internal medicine—next year adding neurology and neurosurgery to the ranks. We are thrilled to attract such clinical diversity, and know that these formidable challenges will necessitate an ‘all hands on deck’ approach within the House of Medicine. As funding streams and training opportunities emerge, it is our aspiration that these early lessons may serve as a replicable template for dissemination and maturation of a critical knowledge base, elevating our clinical workforce to meet the complex demands of this emerging health crisis.

The White House Office and Domestic Climate Policy and the National Climate Task Force have stated interest in a whole-of-government approach to the climate crisis. The whole-of government approach includes representatives from 21 federal agencies and departments deemed essential to the response to climate challenges in the US. The fellowship currently targets preceptorships with federal agencies with traditional mandates toward climate and health. However, a whole-of-government approach to the climate crisis acknowledges an increased need and opportunity for connections with other agencies not traditionally operating in the climate and health sphere. As such, we envision future climate and health fellowship preceptorships within the Department of Energy, Department of Transportation, Department of Housing and Urban Development, and more that can help facilitate cross-sectoral solutions to meet the objectives of our Domestic Climate Policy.

In 2022 Fellows will be supporting the activities of the National Academy of Medicine’s (NAM) Grand Challenge on Climate Change and Human Health. This initiative is a “multi-year global initiative to improve and protect human health, well-being, and equity by working to transform systems that both contribute to and are impacted by climate change,” and each of the five fellows will be participating in member deliberations and activities of this NAM program (18).

For future fellowship cohorts, professional infrastructure is being built to facilitate enhanced state-level and local-level engagement in policy and advocacy. Fellows will connect with policymakers and medical organizations in Colorado as the identified climate and health experts in the region, advocating for and shaping climate action policy. Fellows will also partner with health policy trainees in other clinical departments at CU to further develop their advocacy skills throughout the year.

## 6 Acknowledgment of constraints

The authors acknowledge that a predominantly remote fellowship designed to accommodate and be tailored to the diverse and unique interests of each fellow comes with limitations. Namely, with a primacy placed on a nimble and adaptive learning environment, assessment of participant outcomes is limited due to the high degree of variation in participant area of focus. Despite this limitation, future fellowship cohorts will benefit from a more robust set of evaluation methods designed to capture their mastery of the competencies described in Table 1.

Finally, fellowship faculty recognize that advocacy in the field of climate and health is strongest when bolstered by an interdisciplinary coalition composed of clinicians from diverse backgrounds. Indeed, physicians are not the only trusted messengers in healthcare. Nurses and other healthcare professionals can also play a key role in advancing the mission of the fellowship and its partners. Incorporating professional diversity is a priority for the program and future iterations of the fellowship will look toward non-physician clinician experts to expand the voice of health professionals in the field of climate and health.

## Data availability statement

The original contributions presented in this study are included in the article/supplementary material, further inquiries can be directed to the corresponding author.

## Author contributions

JL and SW: study conception and design. SW and BC: draft manuscript preparation. All authors reviewed the results and approved the final version of the manuscript.
